# Commentary: Exploring the mental healthcare needs of Swiss pre-trial detainees: A pilot investigation of an on-site psychiatric day clinic

**DOI:** 10.3389/fpsyt.2022.1004416

**Published:** 2022-10-04

**Authors:** Michael Liebrenz, Roman Schleifer, Alexander Smith, Tania Urso

**Affiliations:** Department of Forensic Psychiatry, University of Bern, Bern, Switzerland

**Keywords:** day clinic setting, pre-trial, criminal justice system, psychiatry and detention, cultural psychiatry

## Introduction

We read with great interest the article by Gerth et al. (1) recently published in this journal about a psychiatric day clinic initiative involving pre-trial detainees in Zurich, Switzerland. We commend the authors for their insightful preliminary data and believe that their results continue debates about day clinics within the criminal justice system and wider forensic settings where evidence remains limited. We have recently examined literature on this topic amidst jurisdictional frameworks (2). In the spirit of advancing scientific dialogue, we highlight several theoretical considerations and suggested directions for future research in this field.

## Discussion

Gerth et al. outline how day clinics offer an economically viable treatment option whilst maintaining clinical outcomes, as general psychiatric literature indicates. The authors discuss their diverse applicability for inpatient treatment, outpatient treatment, community environments, and long-term care.

Within the criminal justice system and the broader forensic-psychiatric discipline, data concerning the implementation of day clinic services is scarce and discussions arise as to where during the continuation of care they are most beneficial. As Gerth et al. affirm, the welfare and care of justice-involved individuals is critical and additional support is necessary for individuals with prior history of mental illness whose symptoms may be exacerbated by pre-trial conditions. Nevertheless, for us, there are specific questions about the applicability of day clinics in intramural detention settings and in our opinion, this includes those in pre-trial or serving a sentence. Consequently, we believe that more research is needed into how the framing of day clinics in the criminal justice system relate to widely-accepted criteria in general psychiatry (where this concept originated). This is necessary to avoid adverse treatment outcomes and potential mislabelling amongst healthcare and legal professionals.

Whilst they may be integrated in many forms, the predominant view in general psychiatry is that day clinic patients “will benefit from remaining in [their] familiar social environment despite comprehensive therapy” (3). In our view, this notion is problematised in intramural settings as the patient is incarcerated, particularly during pre-trial conditions where a detainee can be potentially exonerated of all charges. Even if the individual is found guilty, pre-trial environments by design are intended to function as a temporary transition period. Akin to Gerth et al., within the precarious environment of pre-trial detention, we very much favor the availability of more intensive psychiatric and psychological support in comparison to typical care provisions; in this regard, Gerth et al. rightly highlight elevated levels of suicidality in such settings. However, we believe that more debate is needed within the scientific community to better define the terminology about this treatment approach in an intramural framework.

In this regard, we envision the implementation of day clinics as extramural in these contexts. This may be appropriate for detainees with mental health disorders as well as for forensic in-patients undergoing a therapeutic measure for whom the step into a residential environment with independent living is too large. Alternatively, it may benefit individuals where available therapy is not intensive enough to accommodate mental health needs in an intended residential facility. Additionally, a day clinic within the criminal justice system and wider forensic framework may be suitable for those who are unable to maintain structured routines or where outpatient therapy is not intensive enough. Given the knowledge-base on outpatient clinics and their effectiveness and shortcomings (4), we believe that day clinics should be accessible, provide regular medical consultations, offer various treatment approaches, include medication dispensation in light of the prevalence of dual diagnoses, and have on-hand physicians who can react should there be a need to transfer back to an inpatient unit. Nonetheless, whilst Gerth et al. conducted their study in Switzerland, it is conceivable that resource availability, for example in developing countries, may problematise and undermine the accessibility of outpatient care and therefore necessitate additional psychiatric support in intramural settings. However, for the reasons we have discussed above, we would again be hesitant to recommend day clinic terminology to describe this scenario.

In other international correctional services, care deficiencies in intramural day clinics have been noted. For example, in France, Fovet et al. illustrated environmental challenges in providing therapeutic care within these services (5). Similarly in Neumünster, Germany, an intramural day clinic was established in 2016. This was designed to accommodate individuals with psychiatric disorders who would benefit most from this setting (6). Nevertheless, the scarcity of full inpatient treatment and resources for detainees with severe mental disorders led to the modification of the triage process (6). Accordingly, individuals who would typically require more intensive inpatient care, like those with schizophrenia spectrum disorders and double/multiple diagnoses, were admitted (6). This limited the clinical effectiveness of the original design (6). Gerth et al. support this notion and do not recommend the admittance of schizophrenic patients in their paper. Yet, as was demonstrated in Neumünster, clinical realities might necessitate the transfer of patients for whom the system was not initially designed.

Interestingly, Gerth et al. found that women did not benefit from their programme, which suggests a need a further for research into gender-based considerations over a longer-time period, as they acknowledge. Furthermore, Gerth et al. note in their limitations that they had no access to data on ethnicity or racial identity. This is significant since difficulties arose in Neumünster in relation to the evaluation and care of patients with limited German-language skills due to a lack of interpreters and language deficiencies in diagnostic tools (6). We wish to emphasize the importance of cultural competency and culturally-appropriate communication in all psychiatric care (7). Moreover, an advantage of day clinics amongst the general population is that they entail lower stigmatization of mental health issues (8). Conversely, the opposite may be true in intramural settings, as research shows that detained individuals with psychiatric disorders face substantial stigmatization (9); special care should be given to mitigate against this, which could also form the basis for additional investigations.

## Conclusion

We commend the authors for their interesting and timely contribution to the debate around day clinics in the criminal justice system. We have suggested directions for future developments in this area and discussed theoretical considerations. Until more evidence is gathered on this topic, it is our view that those in the field should be careful about the terminology and framing of day clinics in intramural settings. This needs to be scrutinized and evaluated further to ensure sufficient mental health treatment is provided to vulnerable detainees.

## Author contributions

ML, RS, AS, and TU conceptualized and drafted the piece. All authors contributed, read, edited, and approved the submitted version.

## Conflict of interest

The authors declare that the research was conducted in the absence of any commercial or financial relationships that could be construed as a potential conflict of interest.

## Publisher's note

All claims expressed in this article are solely those of the authors and do not necessarily represent those of their affiliated organizations, or those of the publisher, the editors and the reviewers. Any product that may be evaluated in this article, or claim that may be made by its manufacturer, is not guaranteed or endorsed by the publisher.
